# Medico-Legal Aspects on Skin Grafting

**Published:** 1887-04

**Authors:** 


### Medical News.

Medico-Legal Aspects of Skin Grafting.—On August the 30th, 1886, in the presence of Drs. Hardon, Westmoreland and Howell, Dr. Henry Wile of this city proposed to a boy of thirteen years to submit to the removal of some small skin-grafts from his arm, to be placed upon an extensive ulcerated surface on the head of his cousin, a little girl somewhat younger, whom he had accompanied to the office. The boy readily consented, and minute grafts were excised without causing him any inconvenience.

In the afternoon of the same day, the father of the boy went to the office and charged Dr. Wile with having cut "his son's arm to pieces." He subsequently swore out a warrant charging him with assault and battery, whereupon Dr. Wile waived examination and gave bond in the sum of two hundred dollars for his appearance at the City Court.

The trial was before Judge Van Epps without a jury, and after reviewing the facts as above given, the Judge stated that the boy had more than ordinary intelligence and discretion, and that a child of his age, under such circumstances of intellectual development, could commit crime and be punished according to law. He considered, therefore, that he had a right to give his consent, so that no crime was committed, and the case was dismissed.—*Atlanta Medical Journal.*

